# A game of snakes and ladders: the world of complex health and social care data linkage

**DOI:** 10.23889/ijpds.v9i5.2823

**Published:** 2024-09-18

**Authors:** Kaat De Corte, Elizabeth Crellin, Freya Tracey, Sarah Hardy, Richard Brine, Therese Lloyd

**Affiliations:** 1 The Health Foundation; 2 Our Future Health

## Objective and Approach

Research on social care services requires large, comprehensive, routine datasets. Launched in November 2019, Developing resources And minimum data set for Care Homes’ Adoption (DACHA) study aims to develop a prototype minimum dataset as proof of concept and to propose implementation.

We describe our experience since 2021 of identifying, applying for, and linking care home, local, integrated care system (ICS) and national datasets, including direct-care software-provider data, GP and NHS England (NHSE) datasets.

## Results

Our key challenges:

1. Risk aversion and fragmented information governance within and between organisations.
2. Complex information governance structures must be understood before participants are recruited.
3. Shifts in the data landscape: data ownership moved from clinical commissioning groups to ICSs; NHSE merged with NHS Digital.
4. Research funding: research is required of ICSs, but it is not a system priority, so processes and funding aren’t in place, and research burdens an already strained system.

Our key lessons:

1. Formalising agreements early on and obtaining, and maintaining, senior, committed buy-in.
2. Studies should value the data sharing process through the inclusion of data controllers and information governance staff in the system in research project budgets.
3. Studies should value the data sharing process through the inclusion of data controllers and information governance staff in the system in research project budgets.

## Implications

The DACHA study overlapped with the COVID-19 pandemic and could only engage a limited number (n=3) of ICSs within project resources. Nevertheless, the complexity of the system and the plurality of actors delay or even block legitimate research interests.

